# The relationship between social creativity and aggressive behavior among Chinese junior high school students: The moderating role of hostile attribution bias

**DOI:** 10.3389/fpsyg.2022.953361

**Published:** 2022-11-24

**Authors:** Feifei Ren, Yuanyuan Sun, Lin Ji, Xing Wei

**Affiliations:** ^1^Department of Psychology, Normal College, Qingdao University, Qingdao, China; ^2^Pingdu Education and Sports Bureau, Qingdao, China

**Keywords:** social creativity, originality, appropriateness, harmfulness, aggressive behavior, hostile attribution bias

## Abstract

Recently, research has begun to pay attention to the dark side of creativity. This research attempted to explore the association between social creativity and aggressive behavior as well as the moderating role of hostile attribution bias. Data were obtained from 496 junior high school students in two cities in China using a convenience sampling technique. The results showed that different aspects of social creativity were different related to aggressive behavior. Specifically, appropriateness was negatively, and harmfulness was positively related to aggressive behavior; However, the relation between originality and aggressive behavior was not significant. In addition, hostile attribution bias moderated the relationships between appropriateness/harmfulness and aggressive behavior. Specifically, the negative relation between appropriateness and aggressive behavior as well as the positive relation between harmfulness and aggressive behavior became non-significant when hostile attribution bias was low. Implications of this study are also discussed.

## Introduction

Creativity, defined as the capability to generate useful and novel ideas (Runco and Jaeger, 2012), plays a significant role in adolescents’ survival and success (Florida, 2002). Creativity is generally considered a positive cognitive ability (Lüdeke et al., 2020; Cheng et al., 2021). However, it is becoming increasingly recognized that creativity might also have dark aspects. On the one hand, researchers have started to focus on creativity that might threaten or harm others, such as malevolent creativity (Cropley et al., 2008; Harris and Reiter-Palmon, 2015; Gao et al., 2022) and negative creativity (Clark and James, 1999; James et al., 1999; Cropley et al., 2014). On the other hand, studies have indicated that creativity is associated with problem behaviors. For example, Cropley et al. (2008) found that creativity was correlated with criminal actions. Gino and Ariely (2012) also observed that highly creative students were inclined to cheat more often than less creative students. Research on the dark side of creativity has broadened the understanding of creativity and has led to a move away from a focus on only the beneficial aspects of creativity.

Research on the dark side of creativity is on the rise, and some studies have begun to focus on the association between creativity and aggression. However, the relationship between creativity and aggressive behavior has been inconsistent or equivocal. Some scholars have argued that creativity can reduce aggression (Lubart et al., 2004). The reason is that when individuals encounter interpersonal conflicts, creative answers can be substituted for “fighting” responses (Mouchiroud and Bernoussi, 2008). However, others have suggested a positive correlation between creativity and aggressive behavior (Saggar et al., 2019; Lüdeke et al., 2020). Highly creative individuals look for creative solutions that benefit themselves (Cropley et al., 2008) and tend to break the rules and law (Petrou et al., 2018). They fear social labeling less, which may enable them to develop and carry out creative ideas to perform aggressive behavior (Lüdeke et al., 2020). Empirical results have also been unclear: studies have found positive (e.g., Tacher and Readdick, 2006; Cheng et al., 2021), negative (e.g., Baquedano and Lizarraga, 2012), and no significant (e.g., Hao et al., 2020) relationships between creativity and aggressive behavior. These arguments and inconsistent conclusions might suggest that additional research is needed to clarify the association between creativity and aggression.

Aggressive behavior is a negative aspect of interpersonal interactions. Research has shown that interpersonal problem-solving skills have a long-term positive impact on an individual’s social adjustment, including enabling children to develop harmonious interpersonal relationships and to effectively resolve interpersonal conflicts (Frye and Goodman, 2000; Gu et al., 2014). Considering the domain specificity of creativity (Li C. et al., 2020), aggressive behavior might be more sensitive to creativity in the interpersonal domain. Therefore, the present study examined the relationship between social creativity, referring to creativity in interpersonal problem-solving, and aggressive behavior.

### The criteria of social creativity

Social creativity is defined as an individual’s capability to generate appropriate and novel ideas when solving interpersonal problems (Mouchiroud and Lubart, 2002; Zhang et al., 2013). Regarding the criteria of social creativity, scholars hold different perspectives. For example, Zhang et al. (2013) investigated elementary school students’ social creativity in terms of fluency, originality, and appropriateness. Gu et al. (2014) indicated criteria of social creativity include five dimensions: novelty, appropriateness, fluency, flexibility, and effectiveness in a sample of 210 adolescents. More recently, using a sample of 34 participants, Wang et al. (2018) evaluated social creativity in terms of originality, appropriateness, and usefulness. Based on the definition of social creativity and the previous studies, originality and appropriateness have always been considered the two fundamental elements of social creativity (Zhang et al., 2013; Gu et al., 2014; Wang et al., 2018). Originality refers to the uniqueness or rareness of a view, and appropriateness is defined as the effectiveness and feasibility of ideas (Runco and Jaeger, 2012).

However, harmfulness as important criteria has been ignored in the evaluation of social creativity. Hao et al. (2016) pointed out that the ideas generated for interpersonal problems might be neutral, benevolent, or even malevolent. That is, harmful ideas might also be generated when solving interpersonal problems. According to the “motivation to be creative” model, a negative disposition with the goal of being creative is likely to produce negative creativity (James and Taylor, 2010). In other words, when driven by negative dispositions, individuals might generate harmful ideas. However, ideational harmfulness has been considered important criteria mostly in malevolent creativity (Cheng et al., 2021; Gao et al., 2022). On the other hand, the effect of harmfulness is usually different from other dimensions. For example, Lee and Dow (2011) examined the relations between creativity assessed with alternative uses tasks and aggressive behavior. They found that ideational harmfulness was positively associated with aggressive behavior, while no such correlation was found in ideational fluency. To provide a more complete and detailed framework of social creativity, it is necessary to consider harmfulness as criteria of social creativity. Thus, the present study evaluated social creativity by assessing the originality, appropriateness, and harmfulness of the ideas generated.

### Social creativity and aggressive behavior

As social creativity is a multidimensional structure, the relationship between it and aggressive behavior may be complex. Yet, there is no direct evidence of the association between the two. Therefore, the present study will examine the relations between social creativity and aggressive behavior in terms of three dimensions: appropriateness, harmfulness, and originality.

As for appropriateness, it represents the extent to which problem-solving is appropriate and feasible (Zhang et al., 2013). Jaffee and D’Zurilla (2003) found that more adaptive problem-solving abilities were negatively associated with children’s aggression in a sample of 117 high school students. Similarly, Blair et al. (2000) found that teaching students specific social skills was associated with lower aggression. In addition, Takahashi et al. (2009) also found that children generating prosocial solutions in realistic situations negatively predicted aggressive behavior. This suggests that appropriateness may inhibit aggression to some extent, and when individuals come up with more appropriate and feasible solutions to interpersonal problems, they tend to avoid harming and attacking others. Therefore, we anticipated that appropriateness would be negatively associated with aggressive behavior.

Several researchers have explored the relationship between harmfulness and aggressive behavior. For example, Lee and Dow (2011) found that students’ harmful ideas in a divergent thinking task were positively related to physical aggression in a sample of university students. Other studies have indicated a positive correlation between the harmfulness and implicit aggression of malevolent creativity in the interpersonal aspects of daily life (Harris and Reiter-Palmon, 2015; Cheng et al., 2021). These findings suggest that harmfulness is positively related to aggressive behavior. Although the above results were found in the contexts of malevolent creativity, the harmfulness of malicious and non-malicious contexts has common in threatening or harming others. Thus, we hypothesized that the harmfulness of social creativity may also have positive correlations with aggressive behavior.

Regarding the relationship between originality and aggression, related research is scarce and the results are inconsistent. A study focusing on originality found a positive correlation between second-grade students’ originality in creativity tests and verbal aggression (Tacher and Readdick, 2006). Yet, Hao et al. (2020) failed to find support for the relations between originality and aggression in malevolent creativity. As the findings on originality are inconclusive, we did not hypothesize the relationships between originality and aggressive behavior.

### The moderating role of hostile attribution bias

Another reason for the inconsistent relations between social creativity and aggressive behavior might be the ignorance of other important factors. The general aggression model (GAM) argues that certain traits predispose individuals to perform aggressive behavior, and hostile attribution bias is one trait type of people who frequently attack others (Anderson and Bushman, 2002). Nasby et al. (1980) defined hostile attribution bias as a tendency for individuals to be more prone to attribute hostile motives to other people’s behaviors in an interpersonal situation. Studies have shown that when individuals have high hostile attribution bias, they focus more on negative cues (Dodge and Newman, 1981) and are more prone to have negative psychological feelings and behavioral reactions (Qi et al., 2020). Therefore, the present research further explored the moderating role of hostile attribution bias in the link between adolescents’ social creativity and aggressive behavior.

**FIGURE 1:**
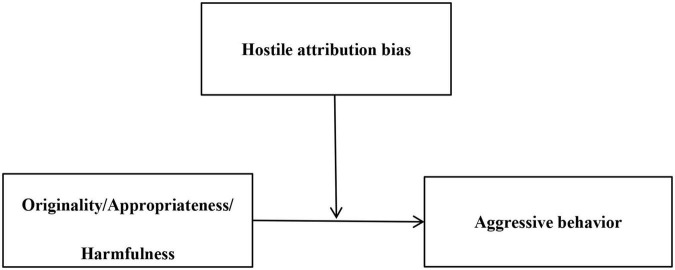
Conceptual model.

Previous studies suggested that hostile attribution bias may be a risk factor. For example, Ellis et al. (2009) found that as hostile attribution bias increased, the association between planning deficits and reactive aggressive behavior increased in a positive direction. Similarly, Osa et al. (2018) proposed that the positive correlation between oppositional behavior and aggression was stronger when children had a high level of relational hostile bias. Li X. et al. (2020) also found that the relation between alexithymia and aggressive behavior was stronger for students with higher hostile attribution bias. One possible reason is that students with a hostile viewpoint of social relations tend to perceive the threat of resource loss (Zhou et al., 2015) and may engage inappropriately, thus predicting subsequent aggressive behavior (Ellis et al., 2009). Thus, according to this “risk” view of hostile attribution bias, when individuals have high hostile attribution bias, the promotion of harmfulness on aggressive behavior will be strengthened, and the inhibitory effect of appropriateness on aggressive behavior will be destroyed. Based on the above reasons, we hypothesized that hostile attribution bias would moderate the association between adolescents’ social creativity and aggressive behavior. Specifically, a high level of hostile attribution bias might strengthen the positive correlation and weaken the negative relationship. The conceptual model is summarized in Figure 1.

## Materials and methods

### Participants and procedures

The sample comprised 496 students from two junior high schools in East China, which contained 265 females and 231 males from grades 7 to 9. Their ages ranged from 12 to 16 years, and the mean age was 13.94 years (*SD* = 0.86). All participants had never participated in similar studies. All participants’ parents gave written informed consent.

### Measures

#### Social creativity

Social creativity was evaluated by offering participants three real-life, open-ended interpersonal problem situations students encounter in their daily lives based on three main contexts of interpersonal interactions (peer, parent-child, and teacher-student situations) (Zhang et al., 2013). It showed satisfactory validity and reliability in the Chinese sample (Zhang et al., 2013; Man et al., 2015). An example of a situational problem is as follows: “Imagine that there is a TV program that you truly want to watch. Imagine that your parents do not agree. What could you do?” Participants were required to come up with as many creative solutions as possible to solve this problem. All problems were scored for originality, appropriateness, and harmfulness of the ideas generated.

Originality refers to the uniqueness or rareness of a view. The original scores were given scores based on their statistical rarity. The opinions given by < 2, 2–4.99, and > 5% participants were respectively given points of “2,” “1,” and “0” (Liu et al., 2013). Appropriateness was defined as the appropriateness and feasibility of the ideas given by participants as a solution and the degree of appropriateness. Harmfulness was defined as the respondents’ degree of malevolence in the solutions. Three well-trained raters evaluated the appropriateness and harmfulness scores independently for each problem for each participant. Appropriateness scores for each problem using a 0–2 scale (0 = not appropriate at all; 2 = very appropriate) (Zhang et al., 2013). Harmfulness scores for each problem using a 1–5 scale (1 = not malevolent at all; 5 = very malevolent) (Gao et al., 2022). The mean appropriateness and harmfulness score for each participant’s views of all three problems were taken as the final appropriateness and harmfulness score. The interrater reliability for participants’ appropriateness and harmfulness scores were higher than 0.94. The Cronbach’s αs were all acceptable in the present study (0.70 for originality, 0.67 for appropriateness, and 0.64 for harmfulness).

#### Aggressive behavior

We assessed each student’s aggressive behavior using 11 items derived from those developed by Crick et al. (1997). The Aggressive Behavior Scale used in the previous study showed satisfactory reliability (Wang et al., 2011; Gasser et al., 2012). It consists of two dimensions: physical aggressive behavior (six items; e.g., “Kicks or hits others”) and relational aggressive behavior (five items; e.g., “Tries to get others to dislike a peer”). Items were rated on a scale ranging from never (0) to always (4), and higher scores represented more aggressive behavior. The fourth question of Crick’s original questionnaire, “Tells a peer that they won’t be invited to their birthday party unless he or she does what the child wants,” was deleted because it was not representative of the Chinese sample in this study (Wang et al., 2011). The reliability of the aggressive behavior scale in this study was 0.90.

#### Hostile attribution bias

Hostile attribution bias was assessed using four items adapted from Krahé and Möller (2004). It consists of two dimensions, such as physical hostile attribution bias (two items; e.g., “Imagine it has been raining for several days and there is water everywhere in the school. At the end of the school day, you and a classmate just walked out of the building, suddenly rushed out from behind a classmate, just stepped on a puddle next to you, splashing you with water”) and relational hostile attribution bias (two items; e.g., “Imagine between classes, you hear two classmates mention your name, and when you walk over to them, they suddenly stop talking”). Sample items are as follows: “You think he or she did it on purpose”; “It makes you very angry”; and “Look for opportunities to screw him or her.” Items were rated on a scale ranging from yes (1) to no (3), and the higher the score, the lower the hostile attribution bias. The hostile attribution bias scale’s internal consistency reliability was 0.85 for this study.

### Statistical analysis

In a preliminary analysis, we examined the alpha reliability of each scale. According to the definition of social creativity, referring to an individual’s capability to generate appropriate and novel ideas when solving interpersonal problems (Mouchiroud and Lubart, 2002; Zhang et al., 2013), we deleted 27 participants whose originality score was 0, 21 participants whose appropriateness score was 0 and 461 participants were finally analyzed. On this basis, we conduct the following statistical analysis. Descriptive statistical analyses were analyzed using SPSS 26.0. Then, regression analysis was used to test the direct effects of social creativity on aggressive behavior. In addition, the PROCESS macro for SPSS was used to analyze the moderating role of hostile attribution bias. Variables that may potentially influence aggressive behavior were added to the model to address any possible confounding.

## Results

### Descriptive statistics

Table 1 reports the means, standard deviations, and zero-order correlations of all variables. The correlation coefficients among social creativity dimensions were *r* = −0.19 to 0.73 (*ps* < 0.001). However, the correlation between originality and aggressive behavior was not significant (*r* = 0.02, *p* > 0.05) or between originality and hostile attribution bias (*r* = 0.04, *p* > 0.05). Appropriateness showed significant negative correlations with aggressive behavior (*r* = −0.16, *p* < 0.01) and hostile attribution bias (*r* = −0.15, *p* < 0.01), and harmfulness was positively correlated with aggressive behavior (*r* = 0.33, *p* < 0.001) and hostile attribution bias (*r* = 0.39, *p* < 0.001). Hostile attribution bias showed a significant positive association with aggressive behavior (*r* = 0.41, *p* < 0.001).

**TABLE 1:** Means, *SD*s, and correlations.

Variables	*M*	*SD*	1	2	3	4	5
1. Originality	3.84	3.04	1				
2. Appropriateness	2.79	1.83	0.73***	1			
3. Harmfulness	1.55	0.65	0.20***	−0.19***	1		
4. Aggressive behavior	0.41	0.61	0.02	−0.16**	0.33***	1	
5. HAB	1.83	0.44	0.04	−0.15**	0.39***	0.41***	1

*N* = 461, **p < 0.01 and ***p < 0.001. HAB, hostile attribution bias.

### The direct effects of social creativity on aggressive behavior

Table 2 presents the direct effects of social creativity on aggressive behavior. Results showed that appropriateness was negatively and significantly linked with aggressive behavior (β = −0.16, *t* = −3.48, *p* < 0.01), which explained 3% of the variance. Harmfulness was significantly positively associated with aggressive behavior (β = 0.33, *t* = 7.42, *p* < 0.001), which explained 11% of the variance. However, the relations between originality and aggressive behavior were not significant (β = 0.02, *t* = 0.45, *p* > 0.05).

**TABLE 2:** Social creativity’s direct effects on aggressive behavior.

	β	*t*	*p*
Originality→aggressive behavior	0.02	0.45	0.65
Appropriateness→aggressive behavior	–0.16	–3.48	**
Harmfulness→aggressive behavior	0.33	7.42	***

Standardized coefficients are reported. *N* = 461, **p < 0.01 and ***p < 0.001.

### Moderating effects of hostile attribution bias in the relations between appropriateness/harmfulness and aggressive behavior

We assessed the moderating role of hostile attribution bias in the relations between appropriateness/harmfulness and aggressive behavior in Table 3. In the first model, gender, one-child status, and grade were introduced as control variables in the regression equation. In the second model, aggressive behavior was set as the dependent variable, appropriateness and hostile attribution bias, harmfulness and hostile attribution bias were respectively set as independent variables. In the third model, aggressive behavior was set as the dependent variable, and the interaction of appropriateness and hostile attribution bias and the interaction of harmfulness and hostile attribution bias were respectively set as independent variables.

**TABLE 3:** Hierarchical regression analyses to assess the effect of hostile attribution bias on the relationships between appropriateness/harmfulness and aggressive behavior.

	Appropriateness→aggressive behavior			Harmfulness→aggressive behavior		
Variables	Model 1	Model 2	Model 3	Model 1	Model 2	Model 3
Step 1: Controls						
Gender	0.18***	0.16***	0.16***	0.18***	0.16***	0.16***
Only child status	–0.08	−0.11**	−0.10*	–0.08	−0.11**	−0.11**
Grade	–0.08	–0.07	–0.06	–0.08	–0.01	–0.01
Step 2: Main effects						
SC		−0.09*	−0.10*		0.19***	0.15**
HAB		0.39***	0.37***		0.34***	0.30***
Step 3: Interactions						
SC × HAB			−0.10*			0.19***
*R*^2^	0.04	0.22	0.22	0.04	0.24	0.27
△*R*^2^	0.04	0.17	0.01	0.04	0.19	0.03
*F*	6.76***	24.90***	21.84***	6.76***	28.12***	27.32***

Standardized coefficients are reported. *N* = 461, *p < 0.05, **p < 0.01, and ***p < 0.001. SC, social creativity; HAB, hostile attribution bias.

The results showed that appropriateness was negatively correlated with aggressive behavior (β = −0.10, *t* = −2.33, *p* = 0.02), whereas hostile attribution bias had a significant positive predictive effect on aggressive behavior (β = 0.37, *t* = 8.62, *p* < 0.001). Additionally, the interaction between appropriateness and hostile attribution bias had a negative predictive effect (β = −0.10, *t* = −2.31, *p* = 0.02) (see Table 3). This finding indicated that hostile attribution bias acted as a moderator in the link between appropriateness and aggressive behavior. Similarly, Table 3 also shows that harmfulness was significantly and positively correlated with aggressive behavior (β = 0.15, *t* = 3.17, *p* < 0.01), and hostile attribution bias had a significant positive predictive effect on aggressive behavior (β = 0.30, *t* = 6.58, *p* < 0.001). Additionally, the interaction between harmfulness and hostile attribution bias had a significant positive predictive effect (β = 0.19, *t* = 4.25, *p* < 0.001). This finding indicated that hostile attribution bias moderated the relations between harmfulness and aggressive behavior.

**FIGURE 2:**
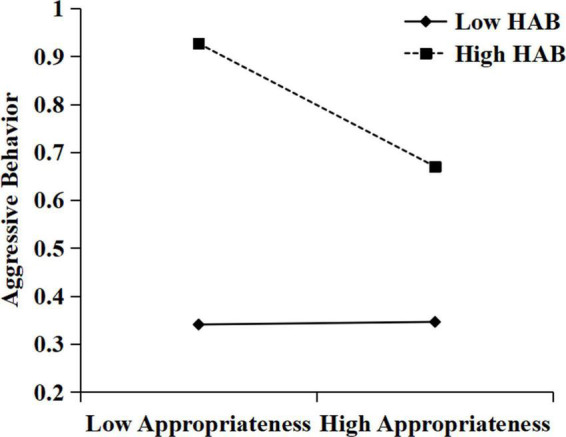
The moderating role of hostile attribution bias in the correlation between appropriateness and aggressive behavior. HAB, hostile attribution bias.

**FIGURE 3:**
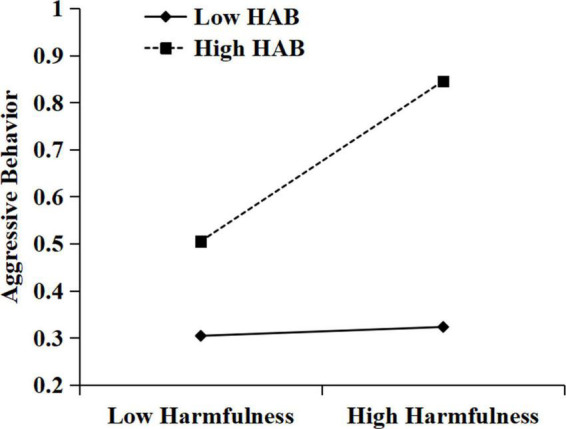
The moderating role of hostile attribution bias in the correlation between harmfulness and aggressive behavior. HAB, hostile attribution bias.

To further analyze the moderating effect of hostile attribution bias, simple slope tests were then conducted. As suggested by Aiken and West (1991), we estimated simple slopes at two levels: high (one *SD* above the mean of hostile attribution bias) and low (one *SD* below the mean of hostile attribution bias). Simple slope analyses indicated that for students with high hostile attribution bias (1 *SD* above the mean), appropriateness was negatively related to aggressive behavior (see Figure 2), simple slope = −0.13, *SE* = 0.04, *t* = −3.15, *p* < 0.01, 95% CI = [−0.20, −0.05]. For low hostile attribution bias (1 *SD* below the mean), appropriateness was not related to aggressive behavior, simple slope = −0.00, *SE* = 0.04, *t* = −0.01, *p* = 0.99, 95% CI = [−0.07, 0.07]. As Figure 3 shows, harmfulness was positively correlated with aggressive behavior when hostile attribution bias was high (simple slope = 0.17, *SE* = 0.03, *t* = 5.56, *p* < 0.001, 95% CI = [0.11, 0.23]). However, harmfulness was not related to aggressive behavior when hostile attribution bias was low (simple slope = 0.01, *SE* = 0.04, *t* = 0.36, *p* = 0.72, 95% CI = [−0.06, 0.09]).

## Discussion

An increasing number of studies have focused on the dark side of creativity. The present research contributes to the literature by examining the relations between social creativity and aggressive behavior as well as the moderating role of hostile attribution bias. The results showed that originality, appropriateness, and harmfulness were related differently to adolescents’ aggressive behavior. In addition, hostile attribution bias moderated the relations between appropriateness/harmfulness and aggressive behavior, and the higher the hostile attribution bias was, the stronger the relationships. Our results partially support the hypothesis proposed.

### The different relationships between appropriateness/harmfulness/originality and aggressive behavior

The present research found that appropriateness had a significant negative correlation with Chinese students’ aggressive behavior. These results agreed with previous findings in Western culture (e.g., Blair et al., 2000; Jaffee and D’Zurilla, 2003). Individuals with high appropriateness might have high interpersonal problem-solving skills and engage in feasible and effective behaviors. They generate adaptive solutions and engage less in aggression when dealing with interpersonal conflict (Lubart et al., 2004). This is especially true in the context of Chinese culture. Influenced by collectivistic values, individuals emphasize more on interpersonal relationships, individual compliance with the collective, and adherence to social norms and standards (Lin, 2009). This may be the reason why Chinese children come up with more appropriate problem-solving strategies. They prefer to have their problem-solving strategies recognized by society and conform to specific social norms so that the strategies or approaches can reduce interpersonal tensions and conflict.

In addition, the present study found that harmfulness was significantly positively correlated with aggressive behavior. This result was consistent with previous research that reported a positive correlation between the harmfulness and implicit aggression of malevolent creativity (Cheng et al., 2021). The study also confirmed the theoretical expectations of the “motivation to be creative” model, which postulated that individuals generate creative ideas that may direct them toward a harmful or malevolent goal (James and Taylor, 2010). Meanwhile, in our study, we found that approximately 71.4% of students had more or less harmful ideas. Therefore, harmfulness is an essential dimension and should be considered when evaluating social creativity. Participants with higher harmfulness scores produced many more malevolent views in solving interpersonal problems and ideas and solutions were often used defensively (Hao et al., 2016). These harmful views may promote destructiveness, making individuals more likely to break social norms (Harris and Reiter-Palmon, 2015), and might contribute to their aggression.

Regarding the relationship between originality and aggression, previous studies have not reached consistent results (Tacher and Readdick, 2006; Hao et al., 2020). This may indicate that the role of originality in influencing aggression is multifaceted. On the one hand, children with high originality but combined with high harmfulness may be more likely to break rules and laws, leading to more delinquent and aggressive behaviors; on the other hand, under conflict situations, children with high originality while combined with high appropriateness may be more capable of generating alternative resolution strategies to avoid aggressive behaviors. These complex differential associations between originality and aggression may be a possible explanation for the absence of a link between originality and aggression found in the current study. This result may also suggest that when examining the relationship between creativity and child outcomes, it is necessary to discriminate the different components of creativity and to consider their joint influence on developmental outcomes.

### The moderating role of hostile attribution bias between harmfulness/appropriateness and aggressive behavior

Guided by the GAM, this research further examined how hostile attribution bias moderated the relations between harmfulness/appropriateness and aggressive behavior. The results showed that hostile attribution bias served as a moderator for the relation between harmfulness, appropriateness, and aggressive behavior. Specifically, harmfulness was more strongly positively related to aggressive behavior when hostile attribution bias was high, and the positive relations became non-significant under low hostile attribution bias. The reason might be that when adolescents have responses shaped by hostile cue encoding, they tend to focus on and adapt harmful ideas to interpersonal problem-solving (Verhoef et al., 2019), thus resulting in aggression. This is consistent with prior studies in which hostile attribution bias might be a risk factor (Lyu et al., 2016; Osa et al., 2018). In contrast, when adolescents had low hostile attribution bias, the positive relation between harmfulness and aggressive behavior was not significant. This suggested that as the level of hostile attribution bias decreased, harmfulness was less likely to trigger aggression. This finding indicated that low hostile attribution bias may work as a protective factor and reduce aggressive behavior among adolescents who hold harmful ideas. Individuals with a low level of hostile attribution bias tend to perceive others as understandable and excusable (Lyu et al., 2016), causing their harmful ideas to remain in the realm of cognition and not be applied in reality.

In contrast with our hypothesis, the results showed that when adolescents had high hostile attribution bias, appropriateness was more negatively related to aggressive behavior, and the negative relations became non-significant under low hostile attribution bias. This implies that appropriateness was effective in inhibiting aggression when individuals had high levels of hostile attribution bias. The reason might be that high hostile attribution bias motivates children who hold feasible and appropriate strategies to act on these positive cognitions, thereby reducing negative behaviors (Ellis et al., 2009). Previous literature also reported that problem-solving skills more strongly predicted adult male violence than hostile attribution bias (Kelty, 2013). In contrast, adolescents with low hostile attribution bias tended to engage less in aggressive behavior; thus, the effect of appropriateness was not significant.

Taken together, our findings suggest that hostile attribution bias plays a different role in the relationships between harmfulness/appropriateness and aggressive behavior. When hostile attribution bias acts as a risk factor, adolescents who hold more harmful ideas may be more likely to act on these ideas and engage in aggressive behavior. This shows that high hostile attribution bias may promote positive relations. On the other hand, when hostile attribution bias acts as an interpersonal “arousal” factor, it motivates or spurs adolescents who hold more appropriate ideas to reduce aggressive behavior. This shows that high hostile attribution bias may also enhance the negative relations. Notably, low hostile attribution bias acts as a protective factor and may weaken the positive relationship between harmfulness and aggressive behavior.

### Limitations and future research

Several limitations occur in this research that should be acknowledged. Firstly, this research was a cross-sectional study used to explore the relationships between social creativity and aggressive behavior, and definitive statements about causality should not be made based on the results of the research. Thus, a longitudinal study is needed to investigate the causal link between social creativity and aggression. Secondly, this study did not find a significant correlation between originality and aggressive behavior. One possible reason might be that the effect of originality on aggressive behavior depends on originality combined with what level of appropriateness and harmfulness. Future research could adopt a person-centered method to test the relationship between different types of social creativity style (the combinations of different levels of originality, appropriateness, and harmfulness) and aggressive behavior. Thirdly, hostile attribution bias was the only potential moderator examined in this research, but other moderators, such as personality and social support, also should be identified and tested in future research.

### Implications

This research contributes to the literature in some aspects. Firstly, the present study adds the harmfulness dimension when evaluating social creativity. The frequency of harmful ideas and the positive effect of harmfulness on aggressive behavior found in our study support the necessity of adding harmfulness. Secondly, this study indicates that different aspects of social creativity have different effects on aggressive behavior, which allows a more comprehensive and clearer understanding of the role of social creativity in adolescents’ aggressive behavior. Our results, to a certain extent, shed light on the existing inconsistent results. Moreover, we identify an important boundary condition between social creativity and aggressive behavior. Our finding indicates that multiple traits of individuals, such as appropriateness and harmfulness of social creativity and hostile attributions, may interact with adolescents’ aggressive behavior.

In addition, this research has certain application significance. First, this study illustrates the impact of appropriateness on reducing aggressive behavior and the role of harmfulness in triggering aggressive behavior. This prompts teachers and parents not only to focus on the beneficial role of social creativity but also to pay more attention to the dark side of creativity. This suggests that they can reduce the occurrence of problem behaviors by enhancing students’ feasibility and appropriateness in interpersonal problem-solving and reducing the production of harmful ideas. Moreover, appropriateness and harmfulness of social creativity and hostile attributions work together in aggressive behavior. Teachers and parents can use cognitive training to reduce adolescents’ aggression. Hostile attribution bias is modifiable (Van Bockstaele et al., 2020). This finding may suggest that training-induced changes in bias may be a useful approach to decrease adolescents’ aggression. Specifically, the ability to generate appropriate ideas in solving interpersonal problems is important when adolescents have high hostile attribution bias. At the same time, it may be a good way to provide positive attribution training to suppress the negative effect of harmfulness on aggressive behavior.

## Conclusion

The present research expands our knowledge about the role of social creativity in adolescents’ aggressive behavior. That is, different aspects of social creativity were different related to aggressive behavior. Specifically, appropriateness was negatively, and harmfulness was positively related to aggressive behavior; However, the relation between originality and aggressive behavior was not significant. Moreover, this research found a moderating role of hostile attribution bias between appropriateness/harmfulness and aggressive behavior based on the GAM. Hostile attribution bias strengthened the negative correlation between appropriateness and aggressive behavior as well as the positive relationship between harmfulness and aggressive behavior.

## Data availability statement

The raw data supporting the conclusions of this article will be made available by the authors, without undue reservation.

## Ethics statement

The studies involving human participants were reviewed and approved by Experimental Ethics Committee of the Department of Psychology of Qingdao University. Written informed consent to participate in this study was provided by the participants’ legal guardian/next of kin.

## Author contributions

FR and YS contributed to the conception, design of the study, and wrote the first draft of the manuscript. FR and LJ organized the database. YS performed the statistical analysis. XW was the holder of the research project and assisted in the statistical analyses. All authors contributed to the manuscript revision and read and approved the submitted version.

## Abbreviations

SC: social creativity
HAB: hostile attribution bias.
